# Toxicological Characteristics of Bacterial Nanocellulose in an In Vivo Experiment—Part 1: The Systemic Effects

**DOI:** 10.3390/nano14090768

**Published:** 2024-04-26

**Authors:** Vladimir A. Shipelin, Ekaterina A. Skiba, Vera V. Budayeva, Antonina A. Shumakova, Alexey I. Kolobanov, Ilya E. Sokolov, Kirill Z. Maisaya, Galina V. Guseva, Nikita V. Trusov, Alexander G. Masyutin, Yanina A. Delegan, Yulia N. Kocharovskaya, Alexander G. Bogun, Ivan V. Gmoshinski, Sergey A. Khotimchenko, Dmitry B. Nikityuk

**Affiliations:** 1 Federal Research Centre of Nutrition, Biotechnology and Food Safety, 109240 Moscow, Russia; antonina_sh@list.ru (A.A.S.); alleexxkl@yandex.ru (A.I.K.); sokolov.iliya1993@gmail.com (I.E.S.); k.maisaya@yandex.ru (K.Z.M.); nikkitosu@yandex.ru (N.V.T.); gmosh@ion.ru (I.V.G.); hotimchenko@ion.ru (S.A.K.); dimitrynik@mail.ru (D.B.N.); 2 Academic Department of Innovational Materials and Technologies Chemistry, Plekhanov Russian University of Economics, 117997 Moscow, Russia; 3 Institute for Problems of Chemical and Energetic Technologies, Siberian Branch of the Russian Academy of Sciences, 659322 Biysk, Russia; eas08988@mail.ru (E.A.S.); budaeva@ipcet.ru (V.V.B.); 4 Department of Biology, Lomonosov Moscow State University, 119991 Moscow, Russia; squiggoth@yandex.ru; 5 Institute of Biochemistry and Physiology of Microorganisms, Federal Research Center “Pushchino Scientific Center for Biological Research of Russian Academy of Sciences”, 142290 Pushchino, Russia; mewgia@yandex.ru (Y.A.D.); kocharovskayaj@mail.ru (Y.N.K.);; 6 Department of Operative Surgery and Topographic Anatomy, I.M. Sechenov First Moscow State Medical University, 119435 Moscow, Russia; 7 Department of Ecology and Food Safety, Institute of Ecology, Patrice Lumumba Peoples’ Friendship University of Russia, 117198 Moscow, Russia

**Keywords:** bacterial cellulose, nanofibers, toxicity, behavioral reactions, liver, intestinal microbiome, rats

## Abstract

Bacterial nanocellulose (BNC) is being considered as a potential replacement for microcrystalline cellulose as a food additive and a source of dietary fiber due to its unique properties. However, studies on the risks of consuming BNC in food are limited, and it is not yet approved for use in food in the US, EU, and Russia. Aim: This study aims to perform a toxicological and hygienic assessment of the safety of BNC in a subacute 8-week administration in rats. Methods: BNC was administered to male Wistar rats in doses of 0, 1.0, 10.0, and 100 mg/kg body weight for 8 weeks. Various parameters such as anxiety levels, cognitive function, organ masses, blood serum and liver biochemistry, oxidative stress markers, vitamin levels, antioxidant gene expression, and liver and kidney histology were evaluated. Results: Low and medium doses of BNC increased anxiety levels and liver glutathione, while high doses led to elevated LDL cholesterol, creatinine, and uric acid levels. Liver tissue showed signs of degeneration at high doses. BNC did not significantly affect vitamin levels. Conclusion: The adverse effects of BNC are either not dose-dependent or fall within normal physiological ranges. Any effects on rats are likely due to micronutrient deficiencies or impacts on intestinal microbiota.

## 1. Introduction

Achieving the goals of sustainable development of civilization requires the development and implementation of innovative consumer products from renewable natural sources. Bacterial nanocellulose (BNC), obtained by the microbial fermentation of waste from processing agricultural plant products, is considered as an attractive alternative to synthetic polymers and wood cellulose in the production of food packaging, cover materials in medicine, composites and coatings, disposable tableware, paper, etc. [1,2]. BNC has a set of unique physicochemical properties, such as a high adsorption capacity and the ability to form stable hydrogels [1,3]; as a result, it is considered as an effective replacement for traditional microcrystalline cellulose (MCC, E460i) as a possible food additive with the functional properties of a thickener, emulsifier, stabilizer, and gelling agent [4]. BNC is proposed as a food ingredient: a source of dietary fiber in the production of low-fat—low-calorie dietary foods [5]. Experiments on animals have shown the ability of certain types of dietary BNC to reduce the accumulation of lipids in the liver that is much more pronounced than that of MCC or fibrillated cellulose of traditional origin [6].

The use of BNC in food production is hampered by a lack of knowledge about the risks of its effects on the human body from long-term dietary consumption. While traditional celluloses, including MCC, fibrillated cellulose, plant-derived nanocellulose and chemically modified celluloses have been extensively studied in vivo in this regard and have received convincing data supporting their safety [7,8], BNC toxicological evaluation was reported in an extremely limited number of research works. Of these, it should be mentioned that an early, subacute study [9] showed the absence of signs of acute toxicity, genotoxicity, and irritation; and showed minimal effect on hematological and biochemical parameters as well as on the weight of the internal organs of rats that received “bacterial cellulose fiber” orally in very high doses (3200–8500 mg/kg body weight (b.w.)). Pinto et al. [10] showed no acute toxicity of BNC after a single oral dose of 2000 mg/kg in rats and also found no evidence of genotoxicity to mouse bone marrow cells after intraperitoneal administration. The biocompatibility of BNC was demonstrated when it was introduced to mice parenterally in the form of porous subcutaneous implants [11].

Unfortunately, most studies were conducted at high doses of nanomaterial (usually more than 1000 mg/kg) which do not reflect possible scenarios of BNC exposure through food products and, in addition, may lead to the masking of possible effects of cellulose fibers due to their high degree of aggregation in the gastrointestinal tract. In a single known study of Hagiwara et al. [12] which used a subacute oral administration of BNC to rats at a “realistic” dose above 5.2 mg/kg body weight (b.w.) per day, an increase was found in the level of serum cholesterol and total bilirubin in males as well as an increase in the weight of the cecum, kidneys, and adrenal glands in females; so, the dose listed above was accepted by the authors as a not observed effect level (NOEL). Thus, data on the in vivo toxicity of BNC were obtained from a small number of studies, some of which included a limited number of analyzed toxicity indicators and used excessively high doses, and not in all cases was the natural route of entry into the body used.

Apparently, as a result of this, BNC is still not approved as a food additive or food ingredient in leading countries around the world. In the European Union, BNC was not listed as an approved food additive as of 2018 [13], and there is no evidence of subsequent revisions to this position. In the USA, according to US FDA resources, BNC, unlike other types of cellulose including chemically modified ones, is not recognized as GRAS (“generally recognized as safe”). The information given in [4,5] on this subject apparently does not correspond to reality. In Russia, BNC is not included in the list of approved food additives. Mechanical transfer to BNC of the permits valid for MCC and fibrillar celluloses [4,5] cannot be justified due to significant differences in the ultrastructural morphology and physicochemical properties of these substances. For example, according to [4], the cation exchange capacity of BNC, amounting to 67.5 mequiv/kg, is more than six times higher than that of plant cellulose of food grades. However, as emphasized in [4], BNC, produced at an industrial scale in Japan and the Philippines, is already exported in large quantities to the USA and European countries for food use.

Therefore, assessing the food safety of BNC and identifying associated risks are an urgent task in food toxicology.

One of the most widespread high-performance technologies for producing BNC is based on fermentation with a symbiotic culture of *Medusomyces gisevii*, known under such traditional names as “Nata” or “Kombucha” and which has a long tradition of food use [14]. BNC obtained by this method has very high content of cellulose allomorph Iα (CIα) in the range of 94–100% and degree of crystallinity (CI) in the range of 88–93%, which will give it high functional characteristics, making it an attractive substance for food use.

The aim of this study is a toxicological and hygienic assessment of the safety of BNC from *M. gisevii* in experiments on rats, by using a complex of neurophysiological, toxicological, biochemical, morphological, and molecular genetic methods.

## 2. Materials and Methods

### 2.1. Samples of BNC and Methods of Its Characterization

A symbiotic culture of *Medusomyces gisevii* Sa-12 was purchased from the Research Institute for Genetics and Selection of Industrial Microorganisms Research Scientific Center “Kurchatov Institute”, Moscow, Russia. *Medusomyces gisevii* includes about 8–10 genera of bacteria, such as *Komagataeibacter*, *Gluconobacter*, *Lyngbya*, *Bifidobacterium*, etc., and 15–30 genera of yeast, such as *Candida*, *Lachancea*, *Kluyveromyces*, *Zygosaccharomeces*, *Schizosaccharomyces*, etc. [15,16].

To obtain the culture inoculum, we used a standard semi-synthetic nutrient medium for the symbiotic culture of *Medusomyces gisevii*, obtained from glucose (CAS No. 5996-10-1 produced by OOO Promsintez, Moscow, Russia) and black tea extract according to [17]. When preparing the nutrient medium, a serving of dry black tea (10 g/L, which corresponds to 3.2 g/L black tea extractives in the medium) was poured with boiling drinking water, then 20 g/L of glucose was added, and the mixture was kept at a temperature of 94–96 °C for 15 min in a closed container, after which it was cooled and filtered through a stainless steel sieve, without sterilization. The inoculum was cultivated under static conditions at a temperature of 27 ± 0.2 °C in a climate chamber (Binder-400, Tuttlingen, Germany) for 7 days. Active acidity was not regulated during the cultivation process, which is optimal for most BNC producers [18].

Sample preparation for sequencing of the symbiotic culture (inoculum), followed by bioinformatic analysis of the results, was carried out according to the following protocol. To isolate DNA, the precipitate (0.25 g) was resuspended into 250 µL buffer (20 mM Tris-HCl (pH 8.0), 2 mM Na-EDTA (pH 8.0)) and 5 µL proteinase K solution (20 mg/mL). The suspension was vigorously mixed on a shaker at a frequency of 225 vibrations per minute and incubated for 30 min in a thermostat at a temperature of 50 °C. Then 350 µL of 240 mM guanidine HCl, 350 µL of 2% SDS, 350 µL of 4% laurylsarcosinate Na, and 400 µL of phenol-chloform mixture (1:1) were added. The suspension was vigorously stirred on a horizontal shaker at a frequency of 225 vibrations per minute for 30 min at room temperature. Then they were centrifuged for 10 min at 13.4 thousand rpm at room temperature in a 1.5 mL Eppendorf tube. After centrifugation, the aqueous phase was selected, and 500 µL of chloroform was added to it. The aqueous phase was collected after centrifugation at 13.4 thousand rpm for 10 min, after which it was precipitated with isopropanol (0.6 volume) at 4 °C for 1 h. The precipitate was collected by centrifugation at 13,000 rpm for 10 min, and the precipitate was washed with cold 80% ethanol before being dried and diluted with water to a volume of 30 µL. DNA preparations were obtained at a concentration of at least 50 ng/µL for three symbiotic culture samples designated as G1, G2, and G3. These samples are inoculates obtained on the 10th day after recultivation for different generations with a difference of one month. Physical DNA fragmentation was performed using ultrasound. The libraries were prepared using the BGI-1000006986 MGIEasy Universal DNA Library Prep Set (MGI, Shenzhen, China), and sequencing was performed using the BGI-1000019859 set (MGI, Shenzhen, China) in an FCL flow cell on DNBSEQ-G50 equipment (MGI, Shenzhen, China). Low quality and short (<80 bp) reads were removed using Trimmomatic ver. 0.39 [19]. The data obtained before and after filtering are presented in Table 1.

Taxonomic classification was performed using kaiju version 1.10.0 https://github.com/bioinformatics-centre/kaiju (accessed on 1 December 2023) [20]. Data visualization was performed using Krona [21], and the database used was Progenomes [22].

BNC biosynthesis was performed in a container with a volume of 441 L; other conditions were similar to those whilst obtaining the inoculum, as a result of which BNC webs were obtained [23]. The samples were sterilized by autoclaving at an excess pressure of 1 atm for 40 min in deionized water, and after sterilization, they were stored hermetically sealed at a temperature of 4 °C.

Characteristics of the BNC samples included determination of the mass of dry substances using a standardized method and sanitary and chemical safety indicators including content of arsenic, lead, cadmium, mercury by atomic absorption spectrometry. Chromatographic multidetection of mycotoxins was performed according to [24]. The ultrastructure of the sample was studied in a JEM 2100 transmission electron microscope (JEOL, Tokyo, Japan) with a Gatan Orius SC200D 2k digital camera (Gatan, Pleasanton, CA, USA) at an accelerating voltage of 200 kV with a LaB6 cathode. Sample preparation involved applying an aqueous BNC suspension to copper grids coated with Formvar film, followed by staining with 2% uranyl acetate and drying. TEM studies were carried out at the Shared Research Facility “Electron microscopy in life sciences” at Moscow State University (unique equipment “Three-dimensional electron microscopy and spectroscopy”).

### 2.2. Experimental Design

The experiment was carried out on 48 male Wistar rats aged 5 weeks, obtained from the Stolbovaya nursery of the Federal State Budgetary Institution Scientific Center for Biology and Medical Sciences of the Federal Medical and Biological Agency of Russia (Stolbovaya, Moscow region, Russia). Care, housing, euthanasia, and experimental procedures were in accordance with international guidelines for good laboratory practices [25]. The experimental design was approved by the Ethics Committee of the Federal State Budgetary Institution of Science “Federal Research Center for Nutrition and Biotechnology”, protocol No. 7 dated 14 October 2022. After a 7-day quarantine, the animals were randomly divided into four groups of 12 individuals. The rats were housed two by two in polycarbonate cages on sawdust bedding. The rats’ initial b.w. in groups 1 to 4 were 123 ± 3; 122 ± 4; 122 ± 4; and 123 ± 3 g (M ± s.e.m; the difference is not significant, *p* > 0.1, ANOVA test). Throughout the experiment, the animals received a balanced semi-synthetic diet according to AIN-93M and drinking water purified by reverse osmosis without restrictions. The rats of the 1st control group were fed with the abovementioned diet without any additives; BNC was added to the diet of the rats from groups 2 to 4, after homogenizing its gel in a blender, in calculated doses of 1.0; 10.0; and 100 mg/kg b.w. for dry substances, respectively. The actual dose of BNC consumed was calculated based on the weight of food consumed daily; if necessary, the amount of BNC added to the diet was adjusted. The total duration of feeding was 56 days (8 weeks).

Daily observation of the animals included a visual assessment of the behavior, activity, condition of the coat and mucous membranes, and stool. B.w. was determined 2 times a week on electronic scales with an error of ±1 g.

The study of the cognitive function of the rats was performed in the “Conditioned Passive Avoidance Reflex” (CPAR) test on the 29th, 30th, and 49th days of the experiment, and the level of anxiety and locomotor activity was assessed in the “Elevated Plus Maze” (EPM) test using equipment from Panlab Harvard Apparatus (Barcelona, Spain). The testing methodology was presented in [26].

The animals were removed from the experiment on day 57 after a 16 h fast by decapitation under ether anesthesia. Blood was taken under aseptic conditions for analysis of biochemical, immunological, and biochemical parameters, and the organs were collected, such as the liver, kidneys, lungs, heart, spleen, thymus, adrenal glands, gonads, small intestine, and brain, and then their mass were determined on electronic scales with an error of ±10 mg.

### 2.3. Analytical Methods

Biochemical parameters of blood plasma (content of glucose, total protein, albumin, globulins, creatinine, urea, uric acid, triglycerides, total cholesterol, and in the composition of low-density (LDL) and high-density lipoprotein (HDL) fractions, alanine (AlAT) and aspartic (AsAT) aminotransferases and alkaline phosphatase activities) were determined on a biochemical analyzer “Konelab 20i” manufactured by Thermo Fischer Scientific (Vantaa, Finland) using reagent kits and methods from the same manufacturer. The content of vitamins A (retinol and retinol palmitate) and E (alpha-tocopherol) in the blood serum and liver homogenate was determined by reverse-phase HPLC with spectrofluorimetric detection [27]. The amount of reduced glutathione in liver homogenates was determined by spectrophotometry using Ellman’s reagent (Sigma-Aldrich, Saint-Louis, MO, USA). The activity of glutathione peroxidase type 1 (GPXI) in red blood cells was measured by the direct spectrophotometric assay with Ellman’s reagent in the presence of sodium azide. The content of catalase in blood serum was determined using an enzyme immunoassay kit produced by Cloud-Clone Corp. (Katy, TX, USA).

The expression of the *Sod1*, *Cat*, *Gpx1*, *Hmox1*, *Nfkb1*, *Bax*, *Nos2*, *Cyp7a1*, and *Hmgcr* genes in the liver was assessed using real-time reverse transcription polymerase chain reaction (RT-PCR) [28]. The primers and probes used were produced by DNA-Sintez (Moscow, Russia). Amplification was carried out on a CFX 96 instrument manufactured by Bio-Rad Laboratories, Inc (Hercules, CA, 94547, USA). Gene expression was calculated based on the threshold cycle value (Ct—cycle threshold) and normalized relative to the housekeeping genes *Actb* and *Gapdh* using the 2^−ΔΔCt^ method.

For histological studies, liver and kidney tissue samples were immediately cooled to a temperature of 0–2 °C after collection, fixed in a 3.7% formaldehyde solution in 0.1 M sodium phosphate buffer pH 7.00 ± 0.05 for at least 3 days, dehydrated in alcohols of increasing concentration, soaked in xylene, and filled with homogenized Histomix paraffin medium at an automated block-filling station. Paraffin sections 3–4 µm thick were prepared on a sled microtome, stained with hematoxylin-eosin, and viewed in an AxioImager Zl microscope (Carl Zeiss, Oberkochen, Germany), equipped with a digital camera at a magnification of ×400 (liver) and ×200 (kidneys). Morphometry was performed using AxioVision Rel.4.8 software (Carl Zeiss, Jena, Germany) and an X/Y calibration slide with a scale division of 10 μm (manufactured by Mikromed, Moscow, Russia).

### 2.4. Statistical Processing of Experimental Data

Statistical processing was carried out using SPSS 20.0 (IBM SPSS Statistics, Armonk, NY, USA) and Microsoft Office Excel 2007. The correspondence of the distribution of values to the normal law was assessed using the Kolmogorov–Smirnov test. In order to increase the stability and convergence of the results, gross errors (outliers of measurement results) of normally distributed values were excluded according to the Grubbs criterion. The number of excluded values did not exceed one in each group. After this, the sample mean M and the standard error of mean s.e.m. were calculated. For indicators with a distribution that did not correspond to normal (behavioral reactions), the median, maximum, and minimum values, and the quartile interval were determined. The significance of differences between the groups was established using one-way analysis of variance ANOVA and the non-parametric Mann–Whitney test as a post hoc test. The significance of differences in alternative indicators was established using the χ-square test. Differences were accepted as significant at the level of *p* < 0.05.

## 3. Results

### 3.1. Microbiome Characterization of Symbiotic Culture Medusomyces Gisevii

The symbiotic culture microbiome is represented by *Proteobacteria*. At the phylum level, its composition is independent of generation, with 99% of reads classified as *Proteobacteria* in all three samples. In 2021, it has been proposed that the phylum *Proteobacteria* be renamed to *Pseudomonadota* [29], but the use of the new term remains controversial among microbiologists [30]. Here, we use the former name of the phylum. Within the phylum, starting from the class level, there are differences in the microbiome of different generations of the *Medusomyces gisevii* (Table 2).

That is, you can see a trend: from G1 to G3, the number of readings corresponding to *Betaproteobacteria* is growing, the number of readings of *Gamma-* and *Alphaproteobacteria* (the latter are already small) is decreasing. *Gammaproteobacteria* is represented by a single genus: *Pseudomonas* (order Pseudomonadales, family Pseudomonadaceae). *Betaproteobacteria* are represented by the genus *Achromobacter* (order Burkholderiales, family Alcaligenaceae). *Alphaproteobacteria* are represented by the genus *Komagataeibacter* (order Rhodospirillales, family Acetobacteraceae) and a number of minor fractions. Thus, according to the sequencing data of the complete metagenome community, the microbiome of the *Medusomyces gisevii* is represented, in fact, by bacteria of three genera. Representatives of the genera *Achromobacter* [31] and *Komagataeibacter* are known to produce bacterial cellulose [32].

### 3.2. Characterization of BNC Sample

**Figure 1:**
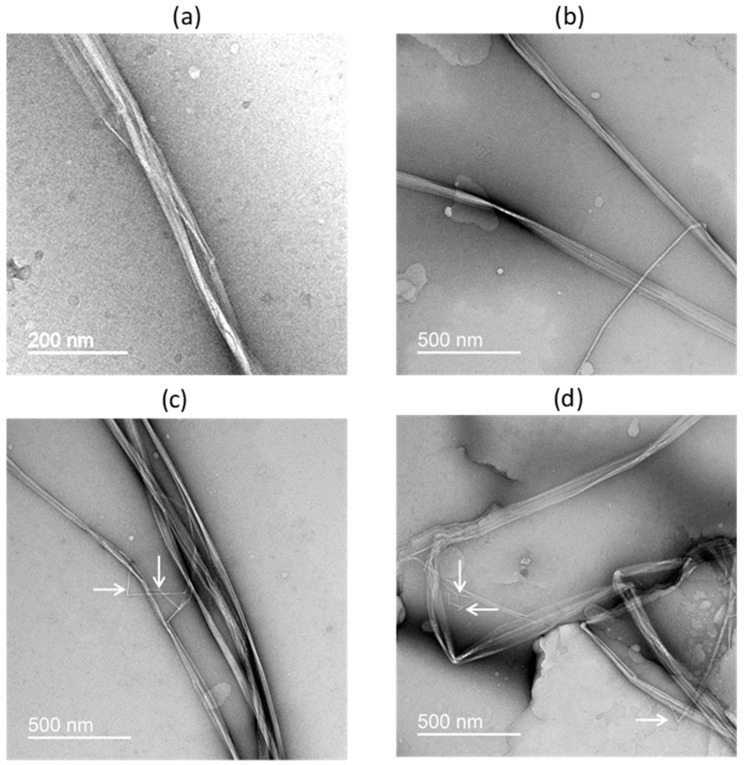
Characterization of BNC ultrastructure by transmission electron microscopy (TEM): (**a**) ultrastructure of an individual BNC fiber (diameter ≈ 50 nm) at high magnification (×100,000): composing nanofibrils with <10 nm in diameter are distinguishable; (**b**–**d**) overview photomicrographs of BNC fiber assemblies at low magnification (×45,000); individual exfoliating nanofibrils (**b**) and, presumably, fragments of nanofibrils (**c**,**d**) are seen, the last indicated by arrows.

According to the results obtained in the Federal State Budgetary Institution of Science “Federal Research Center for Nutrition and Biotechnology” testing laboratory center, the BNC samples corresponded to the sanitary and chemical safety indicators established in Commission Regulation (EU) No 231/2012 of 9 March 2012 in application to MCC of the plant origin food additive (E460(i)), in terms of the content of toxic elements (arsenic, cadmium, lead, and mercury). The method of chromatographic multidetection in the BNC preparation showed the absence within the sensitivity of the analysis of 24 mycotoxins, including aflatoxins B1, B2, G1, sterigmatocystin, ochratoxin A, T-2 and NT-2 toxins, deoxynivalenol, zearalenone, fumonisins B1 and B2, fusarenon X, 3-acetyl- and 15-acetyldeoxynivalenols, nivalenol, citreoviridine, altenuene, citrinin, α- and β-zearalenols, alternariol and its methyl ester, tentoxin, and T-2 triol. A study of the ultrastructure of BNC using the TEM method, showed the material to be composed of fibers with a length obviously greater than 10 μm and an average diameter of 50 nm, with a clearly visible internal structure represented by parallel nanofibrils with a diameter of 5–10 nm or less (Figure 1a). In some micropreparations, the detachment of individual nanofibrils from the fiber was visible (Figure 1b) and the formation, presumably, of short fragments of nanofibrils with a length of 50–150 nm (Figure 1c,d). Thus, the presented BNC sample can be considered as a nanomaterial according to ISO/TS 80004-2:2017 [33].

### 3.3. Integral Indicators in a Subacute 8-Week Experiment

**Figure 2:**
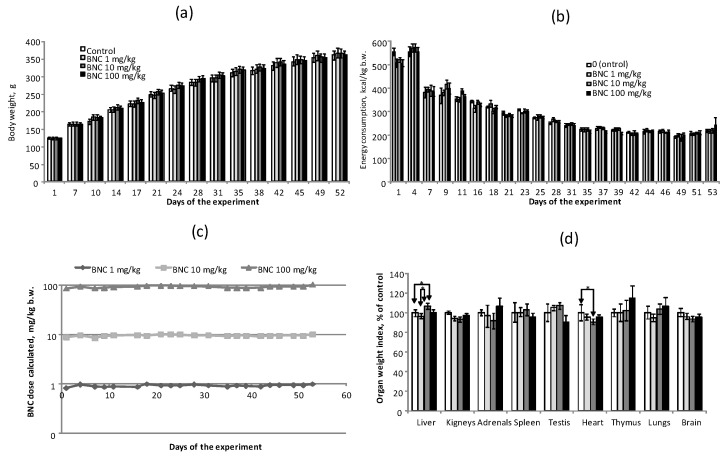
Integral indicators of rats in the subacute oral toxicity experiment. (**a**) Changes of body weight during the experiment; (**b**) the total calorie intake of rats from the diet; (**c**) the estimated dose of BNC consumed by rats with the diet; (**d**) relative (in % of control) mass of internal organs of rats when withdrawn from the experiment. The number of animals—12 per group. * The difference is significant for groups connected by an arrow, *p* < 0.05, Mann–Whitney test.

Throughout the entire period of feeding with experimental diets, the rats of groups 2–4, receiving BNC at calculated doses of 1.10 and 100 mg/kg b.w., had a normal appearance, were active, and had no signs of morbidity observed, and there was no mortality among animals in the experimental groups, with the exception of one rat in group 3 with symptoms of bilateral pneumonia. As follows from Figure 2a, the rats in the control and all experimental groups achieved their b.w. at almost equal speed. At the same time, the specific energy consumption (kcal/kg b.w.) of the animals receiving experimental diets decreased as their b.w. increased (Figure 2b) until the end of the 6th week of the experiment, with subsequent stabilization and no differences between the experimental groups and the control (*p* > 0.1, ANOVA). The decrease in specific energy expenditure observed in all groups is apparently a consequence of a decline in the intensity of energy metabolism due to a decrease in the specific body surface area and an increase in fat mass. Despite all this, the calculated actual consumed doses of BNC were stably maintained in all groups at the initially specified level (Figure 2c).

When the rats were removed from the experiment, two animals from group 3, one from groups 1, 2, and 4 each had bilateral pneumonia (*p* > 0.1 compared to the control for all experimental groups, χ-squared test); no other specific changes were detected in the internal organs. Determination of the relative weight of the internal organs (Figure 2d) showed a small absolute value (6–10%), but a statistically significant decrease in heart weight and an increase in liver weight in group 3 rats (BNC, 10 mg/kg). No other differences in the mass of internal organs were identified.

### 3.4. Influence of BNC on the Behavioral Reactions in Rats

**Figure 3:**
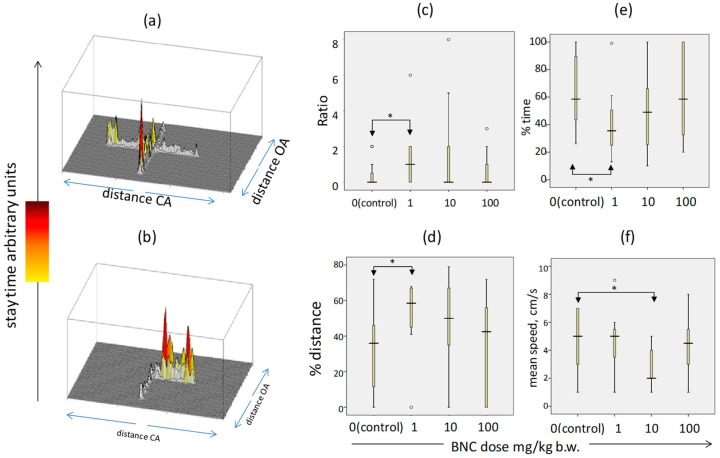
Behavioral responses of rats in the elevated plus maze (EPM) test. (**a**,**b**) Representative profiles of behavior in the maze of animals from groups 1 (control) and 3 (BNC, 100 mg/kg); (**c**) the ratio of residence time in closed (CA) and open (OA) arms of the maze; (**d**) distance traveled in CA, % of total; (**e**) time spent in OA, % of total; and (**f**) average travel speed in CA, cm/s. The number of animals—12 per group. Boxplot charts show the median value (horizontal bar), quartile range (box), range of change (vertical bars), and outliers (circles). * See Figure 1.

When the rats of the control group were placed in the EPM setup, they spent approximately the same time in the open (OA) and closed (CA) arms of the maze (Figure 3a), while the animals of experimental groups 2 and 3 moved mainly in CA (Figure 3b). As shown by a quantitative analysis of the movement of the rats in the installation, the ratio of time spent in CA and OA, which characterizes the degree of anxiety of the animals, was significantly (*p* < 0.05) increased compared to the control in group 2 (BNC, 1 mg/kg) and at the level of a trend (*p* < 0.1) in group 3 (10 mg/kg) (Figure 3c). In group 2, the distance traveled in CA also significantly increased (Figure 3d) and the proportion of time spent in OA decreased (Figure 3e). Group 3 rats moved significantly slower within the CA (Figure 3f). Taken together, this indicates an increase in anxiety in the animals receiving BNC at low and medium doses, but not at the highest dose.

**Figure 4:**
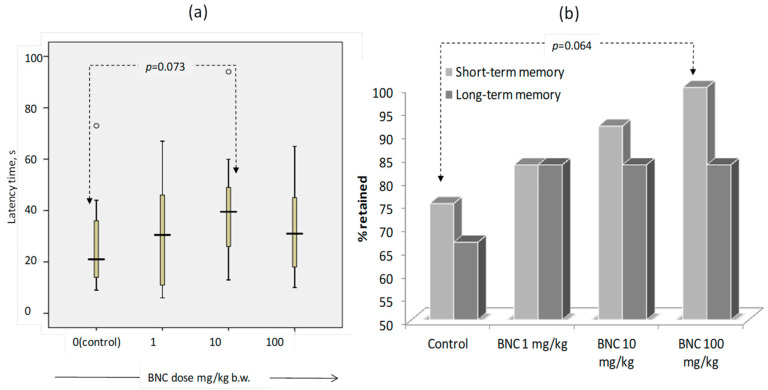
Results of CRPA testing in the rats. (**a**) Latency of the 1st entry into the dark section, and (**b**) % of animals that retained short-term (1-st day) and long-term (21-st days) memory. The number of animals—12 per group. See Figure 3 for boxplot (a) notation. The arrows (dotted line) show the groups that differ at the level of tendency, the *p* value is indicated according to the Mann–Whitney (**a**) and the χ^2^-test (**b**).

During the first testing in the CPAR installation (development of a conditioned reflex), the rats of group 3 were characterized by increased latency time before entering the dark compartment of the installation at the level of a trend (*p* = 0.073) (Figure 4a), and the rats of group 4 (BNC, 100 mg/kg) showed somewhat increased (from 75% in the control to 100%) degrees of short-term memory retention (*p* = 0.064, χ-square test). There was no effect of BNC on long-term (21-day) memory (Figure 4b). Taken together, the findings suggest a comparatively weak effect of BNC consumption on cognitive function in rats.

### 3.5. Blood Biochemical Indicators and the Indices of Fat-Soluble Vitamins Safety

**Figure 5:**
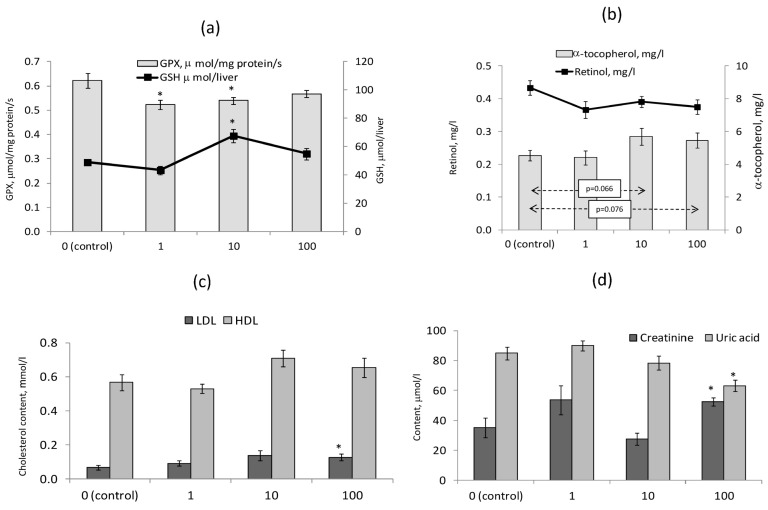
Biochemical parameters of blood and liver of rats. (**a**) Activity of erythrocyte glutathione peroxidase, µmol/min/mg of protein, and content of reduced glutathione in the liver, µmol. Content in blood serum (**b**) of α-tocopherol and retinol, mg/kg; (**c**) of high- and low-density lipoprotein cholesterol, mmol/L; and (**d**) of creatinine and uric acid, µmol/L. The number of animals—8 per group. * Difference with the 1st group (control) is significant, *p* < 0.05, Mann–Whitney test.

In rats receiving BNC at doses of 1 and 10 mg/kg (groups 2 and 3), the activity of erythrocyte glutathione peroxidase was slightly (by 13–16%) but significantly (*p* < 0.05) reduced; at the same time, in group 3 there was an increased content of reduced glutathione in the liver (Figure 5a). In the same group, as well as in group 4 (BNC, 100 mg/kg), there was a tendency (*p* < 0.1) towards an increase in the content of α-tocopherol in blood serum, while the content of retinol practically did not differ from the control in all experimental groups (Figure 5b). The content of both fat-soluble vitamins in the liver also did not differ significantly. Thus, BNC, in any case, does not worsen the safety of both fat-soluble vitamins and does not cause a significant decrease in the studied indicators of the antioxidant defense system.

Biochemical analysis of rat blood serum showed a significant increase in group 4, at the maximum dose of BNC, in the content of LDL cholesterol (Figure 5c) and creatinine (Figure 5d), and a decrease in uric acid. There were no differences between the experimental groups and the control in other indicators, including the levels of glucose, urea, triglycerides, total and HDL cholesterol, ALT and AST activity and their ratios, alkaline phosphatase activity, catalase content, and serum protein and its fractions.

### 3.6. Morphological Indicators and Gene Expression in the Liver

**Figure 6:**
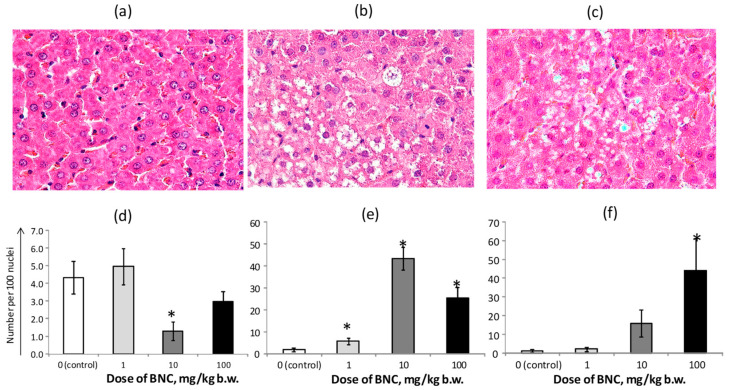
Histomorphological parameters of rat liver parenchyma. Micrographs of liver sections: (**a**) a rat from the 1st group; (**b**) a rat from the 3rd group; and (**c**) a rat from the 4th group. Number per 100 nuclei: (**d**) binucleated cells, (**e**) vacuolized hepatocytes, and (**f**) large vacuoles (balloons). The number of analyzed images—16 (4 from each of 4 rats in the group). * The difference with the 1st group (control) is significant, *p* < 0.05, Mann–Whitney test. Staining with hematoxylin-eosin magnification ×400 (**a**–**c**).

In the rats of the control group, the liver tissue was characterized by a generally normal structure with round hepatocytes, spherical nuclei, a small number of resident macrophages (Kupffer cells), and moderately expressed mitotic activity (Figure 6a). The structure of the liver tissue in rats of group 2 differed only slightly from the control, while in group 3 (Figure 6b) there was a significant decrease in the number of binucleate cells (Figure 6d), indicating a decrease in mitotic activity. In the same group, the number of hepatocytes with small vacuoles (presumably fat droplets) in the cytoplasm significantly increased (Figure 6b,e), while the tissue in group 4 manifested the appearance of large round vacuoles without a discernible internal structure (Figure 6c,f).

A light-optical study of the glomerular and tubular zone of the kidneys showed a normal tissue structure in the animals of all experimental groups. Morphometric analysis did not reveal significant differences between the experimental groups and the control in terms of the size of the glomeruli and Shumlyansky–Bowman capsules, or the degree of their elongation (ellipticity). The only significant difference was a decrease in the ratio of average capsule to glomerular diameter in group 3 (BNC, 10 mg/kg), measuring 1.17 ± 0.05 (M ± s.e.m.) compared to 1.24 ± 0.02 in the control (*p* < 0.05). The same average decrease in group 4 was statistically insignificant.

**Figure 7:**
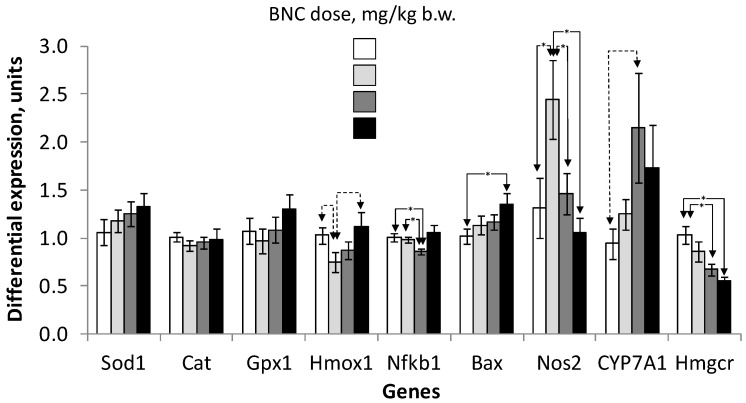
Expression in rat liver of genes associated with metabolic pathways of antioxidant defense and apoptosis. Designations: *Sod1*–superoxide dismutase type 1 (Zn, Cu—dependent); *Cat*, catalase; *Gpx1*, glutathione peroxidase type 1; *Hmox1*, heme oxygenase type 1; *Nfkb1*, nuclear factor kappa b, subunit 1; *Bax*, Bcl2-associated X-type factor; *Nos2*, type 2 nitric oxide synthase; *CYP7A1,* cytochrome P450 family 7 subfamily A member 1; *Hmgcr*, 3-hydroxy-3-methylglutaryl-CoA reductase. Symbols (*, arrows)—see Figure 2 and Figure 4.

Data from PCR analysis of gene expression in liver tissue (Figure 7) showed a decrease (at a trend level) in the level of heme oxygenase-1 (*Hmox-1*) mRNA with a pronounced and significant increase in the expression of nitric oxide synthase type 2 (*Nos2*) in group 2. At the same time, there was a significantly reduced expression of the 1st subunit of nuclear transcription factor Nf-kb (*Nfkb1*) in group 3, and increased expression of apoptosis factor Bcl-2 associated X (*Bax*) in group 4. The maximum level of *Cyp7a1* expression was observed in group 3, although the difference with that of the control was not statistically significant. The expression of *Hmgcr* decreased steadily with increasing BNC doses, with a significant difference between the 3rd and 4th groups from the control.

No differences in the expression of antioxidant enzyme genes (*Sod1*, *Cat*, *Gpx1*) in the liver were detected.

## 4. Discussion

It was shown that BNC in the dose range from 1 to 100 mg/kg b.w. had various effects on the body of rats, although not all of these effects could be interpreted as harmful (toxic), and for a number of studied indicators, there was no monotonic dose–effect relationship, the presence of which is a necessary condition for establishing the NOEL value. The limitations of this study were, firstly, the relatively narrow range of BNC resulting from the method of its introduction into the diet. The amounts of BNC above 100 mg/kg b.w. taking into account the extremely high degree of hydration of cellulose gels would make the diets unpalatable to animals and consequently diminish the nutritional value of feed. On the other hand, these doses of BNC were more consistent with possible food exposure scenarios than the extremely high doses used in the early studies [9,10]. This in some cases made it difficult to compare the results with those studies where significantly higher daily doses were used. Secondly, a number of indicators had not be included in the study design initially (such as the level of vitamin D supply), of which their significance became obvious only after the completion of the experimental data analysis, as will be discussed below.

The identified statistically significant effects of BNC included an increase in the anxiety of the animals in the EPM test at a dose of 1 mg/kg b.w. (which was not confirmed at higher doses) and a decrease in the activity of glutathione peroxidase (GPXI) of erythrocytes at 1 and 10 mg/kg. In animals receiving BNC at a dose of 1 mg/kg, there was also a decrease in the expression of the heme oxygenase I gene (*Hmox1*) in the liver and an increase in nitric oxide synthase type 2 (*NOS2*), which, together with data on GPX activity, may indicate the development of a moderate degree of oxidative stress. The reason for this may be the activation of certain cell populations that react with the release of large amounts of active oxidants, similar to what occurs when plant-derived nanocellulose acts on A549 cell culture [34]. In a study [35], upon oral administration of sulfonated nanocellulose to rats in doses of 50–75 mg/kg, an increase in the expression of inducible NOS2 and apoptosis protein p53, which is a sensitive biomarker of oxidative stress, was observed in kidney tissue, in combination with “moderate cortical congestion, the presence of interstitial hemorrhages and protein casts in the kidney tubules”. Finally, potentially unfavorable and dose-dependent effects include an increase in the number of liver hepatocytes with the presence of cytoplasmic vacuoles at a BNC dose of 10–100 mg/kg and manifestations of balloon degeneration of liver tissue at 100 mg/kg, which presumably may indicate the development of fatty hepatosis.

There is no information in the available literature on the effect of oral administration of nanocelluloses on the level of anxiety in experimental animals, so these effects were identified by us, apparently for the first time. Since the possibility of penetration of very long BNC nanofibers from the gastrointestinal tract into the brain seems extremely unlikely, it should be assumed that there is an indirect mechanism for these phenomena associated with the effect of metabolites of intestinal microflora on the brain [36,37], which is the putative target of BNC. The study of BNC influence on the intestinal microbiome of animals is the subject of our subsequent publication.

As one of the mechanisms of the adverse health effect of nanocelluloses, the literature considers a decrease in the bioavailability of various nutrients (zinc, iron, fat-soluble vitamins) to be due to their binding to cellulose nanofibers that are not absorbed in the intestine and poorly metabolized by microflora [38,39]. There is known data on the ability of certain types of celluloses of plant origin to inhibit the intestinal absorption of vitamins A and E [40]. In our study, however, it was shown that BNC in the applied dose range, in any case, does not impair the safety of vitamins A and E in animals receiving the balanced diet. Unfortunately, the analysis of the effect of BNC on the bioavailability of vitamin D for rats was beyond the scope of this study. As has been repeatedly shown in clinical observations, deficiency of this vitamin is an important etiological factor in the development of non-alcoholic fatty liver disease [41] and metabolic syndrome [42]; in particular, the latter is reflected in the growth of LDL cholesterol levels in agreement with our observations (see Figure 5c). Deficiency of vitamin B_12_ [43] and lipoic acid [44,45] can also disturb lipid metabolism and lead to excess fat accumulation in the liver; this is considered the initial study of steatohepatitis. The accumulation of fat in the liver, most noticeable at the highest dose of BNC in group 4, could have a negative impact on hepatic metabolic function in terms of the transformation of nitrogenous compounds, as reflected by an increase in creatinine levels and a decrease in uric acid in the blood serum; the latter could also result in a decrease in blood antioxidant activity.

At the same time, in studies [46,47] over a wide range of consumed doses, oral intake of various types of both chemically modified and unmodified nanocelluloses of plant origin did not lead to any pathological changes in integral, biochemical, hematological, and immunological parameters (including the level of production of pro-inflammatory cytokines), with the exception of the observed signs of “vacuolization of periportal liver hepatocytes”, which is consistent with the histological changes identified in our study. It should be noted that the authors of the works [46,47] did not consider the morphological changes in the liver tissue they identified as significant signs of toxic effects. This opinion may be based on the fact that the spontaneous appearance of a certain number of vacuoles in the periportal region of rat liver tissue occurs quite often in healthy animals of a given species, sex, and age [48] which apparently does not go beyond the physiological norm.

In this regard, it can be noted that changes in the biochemical parameters of blood serum characterizing the metabolic function of the liver were absent or small in absolute value (creatinine, uric acid, LDL), also within the range of normal values. Analysis of the hepatic expression profile of genes involved in cholesterol synthesis and metabolism showed that changes in *Cyp7A1* expression levels closely resembled the same hepatocytes number with fatty inclusions (compare Figure 6e and Figure 7). However, the lack of statistical significance of these changes does not allow us to unequivocally state the intensification of cholesterol oxidation processes in groups 3 and 4 under the influence of this enzyme. As for the expression of *Hmgcr*, we can confidently assume that the expression of this gene does not allow us to consider the observed signs of liver vacuolization in groups 3 and 4 as the initial stages of the development of non-alcoholic steatohepatitis, of which this enzyme is a sensitive marker [49]. However, it cannot be excluded that the hypothesized effect of BNC on the supply of vitamin D and some other micronutrients not identified in this study could be the cause of the observed changes in the liver of the animals.

The various effects produced by BNC in the present study, particularly in relation to behavioral responses, were characterized by a non-monotonic dose–response pattern, that is, they were most pronounced at a low (1 mg/kg) or intermediate (10 mg/kg) dose of the nanomaterial, but not at the highest one. Therefore, these indicators cannot be unequivocally taken into account when estimating NOEL. The same non-monotonic nature of the dependence on the dose of nanocellulose administered orally to rats was revealed in [35] for indicators of mineral metabolism and the activity of antioxidant factors in the kidneys. The highest level of reduced glutathione was observed in this work at an intermediate dose of nanocellulose, which is qualitatively consistent with the data of the present study. The absence of an unambiguous dose–response relationship is a general problem in the toxicology of nanomaterials and is associated, according to modern ideas, with the fact that the highest levels of free nanoobjects with toxic or other biological effects are achieved at low or intermediate, but not at high, doses, when the processes of formation of large, biologically inactive particle aggregates become predominant [50] due to the law of mass action. In light of this, the data of works [9,10] receive additional explanation, in which no significant effects of BNC on the health of animals were detected after food use. It is possible that at lower doses, different effects of BNC may nevertheless be observed.

## 5. Conclusions

Thus, this study showed that when administered orally to rats for 8 weeks, BNC at a dose of 100 mg/kg b.w. has potentially unfavorable, dose-dependent changes in the morphology of liver tissue (vacuolization of periportal hepatocytes and balloon degeneration) in combination with moderate shifts in certain indicators of lipid and nitrogen metabolism. A number of effects associated with the oral administration of BNC to the body were characterized by the absence of a clear dose–effect relationship, which to a certain extent complicates the toxicological characterization of this nanomaterial.

The presented data allow us to conclude that the use of BNC in food packaging is safe; however, the possibility of its direct use in novel foods meets certain objections associated with some adverse effects that are presented mainly at the maximum dose of BNC in the diet. There are reasonable grounds to believe that these effects are of a marginal nature, slightly exceed the normal range of age-related changes in animals, and are secondary to the effect of BNC on the safety of certain micronutrients (not clearly established in this study). If this occurs, then such influences can be compensated when using BNC in food products, through fortification with missing nutritional factors. Such fortification will allow maximum use of the beneficial properties of BNC in functional food products and products for special dietary needs.

## Figures and Tables

**Table 1:** Received and processed data after sequencing.

	G1	G2	G3
Reads received (number per file)	2,817,576	2,100,066	776,416
Both surviving	2,084,829 (73.99%)	1,447,380 (68.92%)	567,571 (73.10%)
Forward-only surviving	15,7254 (5.58%)	118,147 (5.63%)	48,302 (6.22%)
Reverse-only surviving	423,014 (15.01%)	399,165 (19.01%)	114,818 (14.79%)
Dropped	152,479 (5.41%)	135,374 (6.45%)	45,725 (5.89%)

**Table 2:** Representation of classes of proteobacteria by reads in different generations of the *Medusomyces gisevii* (% of reads of all *Proteobacteria*).

	*Alphaproteobacteria*	*Betaproteobacteria*	*Gammaproteobacteria*
G1	14	21	65
G2	7	49	42
G3	3	81	15

## Data Availability

Data available on request due to restrictions, e.g., privacy or ethical.
